# Effect of vacuum–release teat versus standard teat use on feeding milestones and breastfeeding outcomes in very preterm infants: A randomized controlled trial

**DOI:** 10.1371/journal.pone.0214091

**Published:** 2019-03-22

**Authors:** Sharon Lisa Perrella, Kathryn Nancarrow, Michelle Trevenen, Kevin Murray, Donna Tracy Geddes, Karen Norrie Simmer

**Affiliations:** 1 School of Molecular Sciences, Faculty of Science, The University of Western Australia, Crawley, Western Australia, Australia; 2 Neonatology Clinical Care Unit, King Edward Memorial Hospital, Subiaco, Western Australia, Australia; 3 School of Mathematics and Statistics, Faculty of Engineering and Mathematical Sciences, The University of Western Australia, Crawley, Western Australia, Australia; 4 School of Population and Global Health, Faculty of Health and Medical Sciences, The University of Western Australia, Crawley, Western Australia, Australia; 5 Centre for Neonatal Research and Education, School of Paediatrics and Child Health, Faculty of Health and Medical Sciences, The University of Western Australia, Crawley, Western Australia, Australia; RCSI & UCD Malaysia Campus (formerly Penang Medical College), MALAYSIA

## Abstract

**Background:**

Breast milk is important to infant health, yet shorter breastfeeding duration is reported for preterm infants. Both breast and bottle feeds are given in the neonatal unit, with full oral feeding often the last milestone to be achieved prior to discharge home. Unlike standard bottle teats, a vacuum release teat requires the application of negative intra-oral pressure to release milk, and so may facilitate breastfeeding in preterm infants. The objective of this study was to determine the effect of vacuum release teat use on timing of achievement of the first full oral feed and on first completion of 24 h full oral feeds. Feeding method at discharge home, 2 weeks, 6 weeks and 12 weeks corrected gestational age were also examined.

**Methods:**

A randomized controlled trial was completed with mothers of preterm infants born 24–33 weeks gestation in the neonatal unit of a tertiary women’s hospital. Infants were randomized to one of two parallel groups using a vacuum release teat or standard teat for oral feeds when the mother was not available to breastfeed. Test weights were completed for all oral feeds. It was not possible to blind participants, care givers and outcome assessors to group assignment due to the nature of the study.

**Results:**

The groups did not differ with regard to timing of achievement of first full oral feed or 24 h of full oral feeds. Significantly more infants in the vacuum release teat group were exclusively fed breast milk at discharge from hospital and breastfed at 3 months corrected gestational age.

**Conclusions:**

Use of a vacuum release teat when the mother is not available to breastfeed may promote preterm breastfeeding skills, resulting in higher rates of exclusivity and longer breastfeeding duration.

**Trial registration:**

The trial is registered with the Australian New Zealand Clinical Trials Registry ACTRN12615000245594.

## Introduction

Human milk is important for the health and neuro-developmental outcomes of preterm infants [1], with a dose-response reported [2, 3]. Most Australian mothers provide expressed milk for their very preterm infants during the hospital stay, and intend to breastfeed for 12 months [4]. However when compared to term infants, the incidence and exclusivity of breastfeeding at discharge and beyond are significantly lower for infants born very preterm [4–6].

Establishment of full oral feeding, that is the ability to suck all feeds by breast and/or bottle feeding, is typically a pre-requisite for discharge from hospital and is often the final milestone to be achieved, thus potentially delaying discharge home. The mother-preterm infant dyad face several challenges in establishing breastfeeding including the infant’s immature sucking dynamics, physical size and co-morbidities, as well as separation from the mother and low milk supply [7, 8]. Further, preterm birth disrupts pre-learning and imprinting of feeding behaviours [9, 10]. While interventions to facilitate bottle feeding have been studied [11], there is limited evidence for strategies to facilitate the establishment and continuation of preterm breastfeeding.

Published evidence indicates that timely establishment of milk production and early feeding practices are important. Delayed initiation of breast expression after preterm birth is associated with lower rates of exclusive breast milk feeding at discharge and shorter breastfeeding duration [12]. Infants that attempt their first oral feeding at the breast are less likely to be fed infant formula at discharge [13], and infants that are fed directly at the breast at discharge have longer breastfeeding duration than those fed expressed breast milk (EBM) by bottle [14].

It is thought that opportunities to practice oral feeding through early and regular contact with the breast may facilitate the establishment of breastfeeding.

However, in settings where mothers are discharged home within days of birth, breast contact is limited by maternal availability. Offering regular oral feeding opportunities from 34 weeks post menstrual age (PMA) is associated with faster attainment of full oral feeding [15], and so bottle feeds are often introduced to progress oral feeding in the absence of the breastfeeding mother. The oro-motor function required to remove milk from a standard bottle teat differs from that of breastfeeding [16]. While there is concern that bottle feeding may impact the development of breastfeeding skills and subsequent breastfeeding outcomes, there is insufficient evidence to guide practice at this time [17].

It is possible that support of oro-motor functions required for breastfeeding may result in earlier achievement of oral feeding milestones. A vacuum-release teat has been developed for preterm infants (Calmita, Medela AG), which releases milk in response to intra-oral vacuum, as occurs during infant suckling at the breast, rather than compression. A previous study compared outcomes for groups of infants using a prototype vacuum-release teat for preterm infants or a standard teat for oral feeds when the breastfeeding mother was not available. Use of the vacuum-release teat was associated with earlier discharge home, but there were no differences in timing of achievement of the first full oral feed or full oral feeds. More infants in the vacuum-release teat group received breast milk at 2, 6 and 12 weeks post discharge, although the differences were not statistically significant [18]. The prototype vacuum-release teat had considerable assembly and cleaning requirements that may have impacted compliance in its use. Further research with a commercially available single use version of the teat (Calmita, Medela AG) that requires no assembly is valuable to confirm the previous findings.

The aim of this study that was to compare the oral feeding milestones and post-discharge breastfeeding outcomes for groups of very preterm infants randomized to use a vacuum-release teat or standard teat during their neonatal unit admission.

## Methods

Participants were recruited from the neonatal unit at King Edward Memorial Hospital, Western Australia. The Ethics Committees of the Women and Newborn Health Service (2014125EW) and The University of Western Australia (RA/4/1/7489) approved the study and parents provided informed written consent.

In this randomized controlled trial participants were allocated to one of two parallel groups with balanced randomization (1:1) to groups using a vacuum release teat (CT) or a standard teat (ST) for oral feeds where the mother was not available for breastfeeding. The trial was registered with the Australian New Zealand Clinical Trials Registry, ACTRN12615000245594, https://www.anzctr.org.au/Trial/Registration/TrialReview.aspx?id=367966&isReview=true

Participants were recruited from 32 weeks PMA. Inclusion criteria were: infants of birth gestational age 24–33 completed weeks who were medically stable at 33 weeks PMA; mothers intending to breastfeed with a total milk expression volume of ≥ 300 mL/24 h at the time of recruitment.

Exclusion criteria included: infants with congenital anomalies, grade 4 intraventricular haemorrhage or periventricular leukomalacia, acute illness and/or infection, continuous positive airway pressure (CPAP) or mechanical ventilation at 34 weeks PMA, and/or mothers with a history of breast surgery.

Initial recruitment was slow in the first 6 weeks, partly due to low neonatal unit admission numbers. Also families that did not meet the geographic or birth gestation study criteria had requested participation. Therefore inclusion criteria were expanded to families living >35km from the study hospital, and the minimum birth gestation was reduced from 28 to 24 completed weeks.

Maternal demographic data were collected as well as parity, previous breastfeeding duration, perceived importance of breastfeeding, 24 h milk production volume and planned breastfeeding duration. Residential address postal codes were used to determine rankings of socio-economic disadvantage according to Australian Bureau of Statistics percentiles [19]. Infant birth gestation and weight, birth weight appropriate or small for gestational age, infant sex, singleton birth, incidence and duration of mechanical ventilation and CPAP, and incidence of late onset sepsis were recorded. Infant PMA, weight and destination were recorded at discharge from the neonatal unit.

This paper reports the clinical outcomes of the randomized controlled trial that compared the effect of vacuum release and standard bottle teat use on the timing of attainment of oral feeding milestones and breastfeeding outcomes (S1 File). These outcomes were measured as part of a study which had the primary aim of assessing total transfer volume (mL) at a monitored breastfeed at 33, 34, 35 and 40 weeks PMA; these results will be reported separately (S2 File).

Allocation to the intervention or control group teat type was randomized when infants reached 33 weeks PMA and were fully enteral fed. A statistician prepared sequentially numbered sealed opaque coded envelopes in blocks of four containing the computer generated treatment allocation for randomization. Recruitment and separate randomization was performed by the researchers for two subgroups of infants; those born 24–27.9 weeks and those born 28–33 weeks gestation. Mothers, rather than infants, were randomized to ensure compliance in cases of multiple birth infants. It was not possible to blind participants, care givers or outcome assessors to the group assignment.

**Intervention group** used a disposable Calmita teat (CT, Medela AG, Baar, Switzerland) for bottle feeds during their stay at the study hospital’s neonatal unit. The functional design of the CT teat is based on breastfeeding biomechanics in that it releases fluid only when negative pressure is applied at or above a set threshold. There is a venting system to prevent collapse of the teat. There are two CT teats of different vacuum thresholds; the Calmita starter teat valve threshold is -10±5 mmHg, and the Calmita advanced teat valve threshold is -30±15 mmHg, similar to that of the Calma teat for term infants (Medela AG, Baar, Switzerland). CT group infants commenced bottle feeds using the ‘starter’ teat and changed to the ‘advanced’ teat after completion of the first full oral feed. The intervention group received Medela Calma teats on discharge from the study hospital to provide the option of continued use of a vacuum release teat for bottle feeds while transitioning to full breastfeeding.

**Control group** used a standard teat (ST, peristaltic narrow neck slow flow teat, Pigeon PTB700, Seoul, Korea, or extra slow flow silicone teat, Grow, Growbaby, Icon Health, Victoria, Australia) for bottle feeds that were given during their stay at the study hospital. These teats release fluid with gravity and when positive and negative pressure, or compression and suction, are applied.

As per standard practice in the study setting, non nutritive sucking and breast contact were encouraged prior to 33 weeks PMA. Daily breastfeeding attempts were commenced at 33 weeks PMA and increased according to the infant’s ability and maternal availability. Bottle feeds were introduced after the infant had demonstrated the ability to latch and suckle at the breast, and were only offered when the mother was not available to breastfeed at a scheduled oral feed. Feeds were limited to 30 min. The following feeding milestones were recorded; PMA at first full oral feed, PMA at achievement of 24 h of full oral feeds, as well as infant weight, PMA and breast milk intake at discharge home. Achievement of a full oral feed ie. transfer of the full prescribed feed volume, was determined by test weighing. Breast milk intake, rather than breastfeeding status, was recorded at discharge as availability of rooming in facilities was limited at the study setting, and mothers were advised to gradually transition to full breastfeeding after discharge home.

Follow up telephone calls were made at 2 weeks, 6 weeks and 3 months corrected gestational age (CGA) with mothers asked to recall details of their infant’s feeding method in the previous 24 h. Infants that received any breast milk, whether by direct breastfeeding or EBM, were classified as having received ‘any breast milk’, while those who received no foods or fluids other than breast milk, by direct breastfeeding and/or EBM, were classified as ‘exclusively breast milk fed’. Infants that received all of their feeds through direct breastfeeding, with no other foods or fluids given, were classified as ‘exclusively breastfed’.

### Statistical methods

The sample size calculation was based on the study’s primary outcome measure of total volume transferred during monitored breastfeeds at 33, 34 and 35 weeks CGA. Sample sizes required to detect a clinically relevant effect of 5mL difference between feeds from full and empty breasts were calculated, indicating that recruitment of 24 infants in each group was needed to have 80% power (Type 1 error rate α = 0.05) of detecting a non-directional difference of this size. The sample size was calculated as if the data will be analysed using an independent samples *t*-test, with actual analyses more powerful, and thus able to detect smaller differences.

After 6 weeks of recruitment, there was concern that the requirement of multiple monitored breastfeeds (ie. from a full and empty breast at 34 weeks PMA, then with and without a nipple shield at 35 weeks PMA) was impacting recruitment so these were replaced with single monitored feeds at 33,34 and 35 weeks PMA. The primary outcome measure of milk volume transferred at the breast, as well as secondary outcome measures including feeding efficiency (mL/min), mean, peak and baseline intraoral vacuum, total sucking duration (min), total suck bursts, ratings of infant breastfeeding behavior, incidence of bradycardia and oxygen desaturation will be reported elsewhere. This paper reports the secondary trial outcomes of timing of achievement of the first full oral feed, full oral feeding and full breastfeeding, and breastfeeding duration.

All recruited participants were considered in this paper. Counts and percentages are provided for categorical variables, whilst means±standard deviations (SD) are provided for continuous variables. Univariate and multivariate Cox proportional hazards mixed models were used to analyse infant PMA at first full oral feed and at achievement of 24 h of full oral feeds. Data were not available for some infants that had been transferred to another neonatal unit prior to achievement of these feeding milestones. Infants that had not achieved these milestones prior to transferring were censored at their PMA at transfer. Univariate and multivariate linear mixed models with individual variance components were used to analyse infant weight and PMA at discharge. Fixed effects considered in the models were group (CT, ST), singleton birth, whether birth weight was appropriate for gestational age (AGA), whether ventilation or CPAP was required, discharge destination (home or transferred to another neonatal unit), birth gestation, maternal 24 h milk production volume at recruitment, and SEIFA percentile whilst the random effect considered was the individual mother. The significance level was set at 5% for the study. Hazard ratios (HR), 95% CIs and p-values are provided for the Cox proportional hazards regression results whilst MDs, 95% CIs and p-values are provided for the linear mixed model results.

Mothers of multiple birth infants did not differ their feeding method between each infant and therefore breastfeeding status was analysed on a mother basis. Specifically, chi-squared tests were used to compare breastfeeding status between groups at discharge as well as 2, 6 and 12 weeks CGA and RRs, 95% CIs and p-values are provided. All statistical analyses were carried out using the R environment for statistical computing [20].

## Results

**Fig 1:**
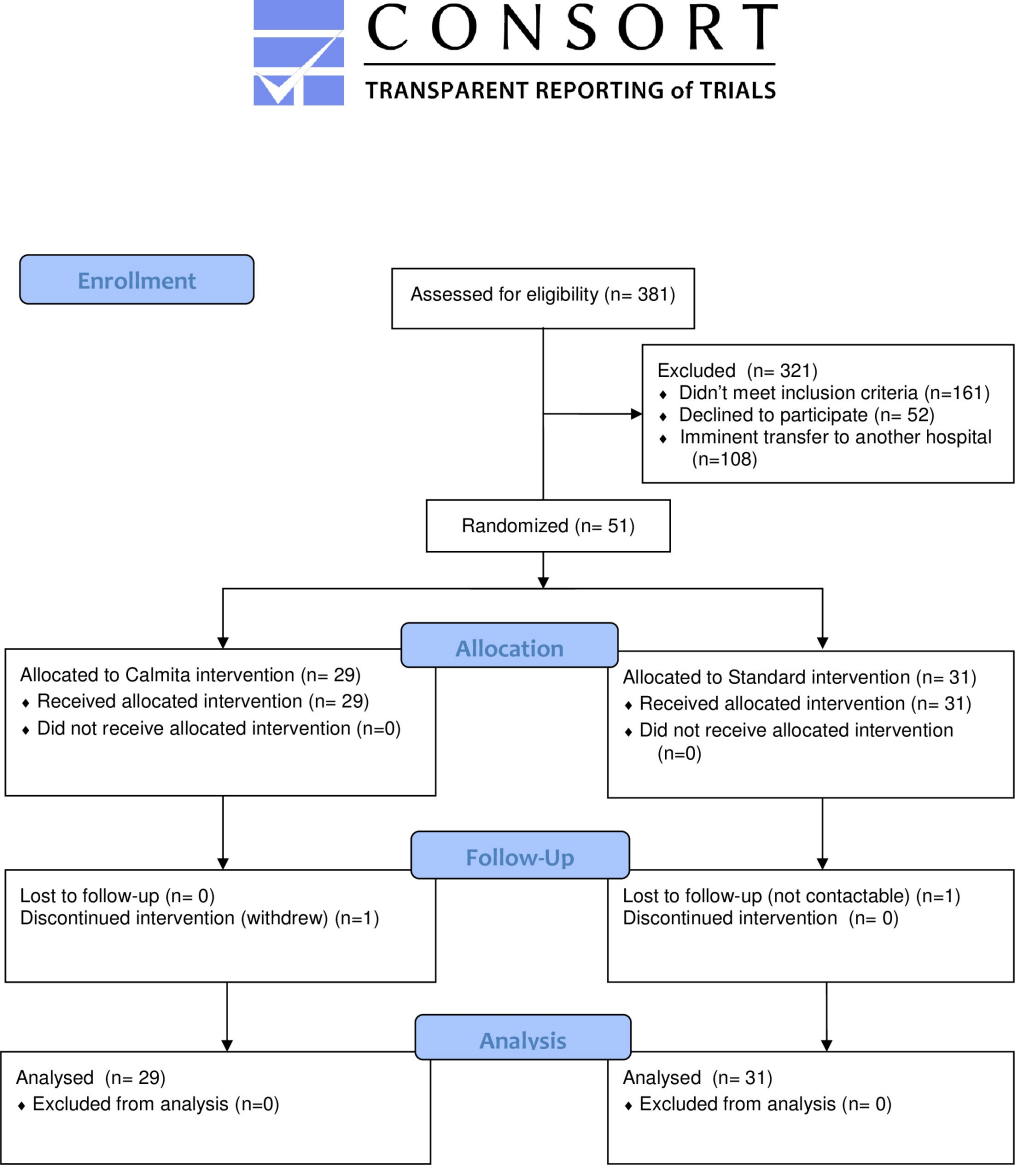
CONSORT flow diagram.

Recruitment commenced September 2015 and was completed in December 2016, with follow-up completed February 2017. Of 381 infants assessed for eligibility, 321 were excluded including 108 due to scheduled transfer to another neonatal unit at the time of recruitment (Fig 1). A total of 51 mothers and 60 infants were eligible and participated in this study, including 10 sets of twins and one twin infant (with the sibling excluded for Grade 4 intraventricular haemorrhage). Thirty-two included infants were transferred to another neonatal unit prior to discharge home. A total of 24 mothers of 29 infants were randomized to the intervention group, and the remaining 27 mothers of 31 infants were randomized to the control group. One set of twins was lost to follow up after transfer to another neonatal unit, and 4 and 2 infants lost to follow up after 2 weeks and 6 weeks PMA respectively.

Table 1 displays maternal and infant characteristics for the entire cohort as well as broken down by treatment arm. Infant characteristics were not different between treatments other than the incidence of mechanical ventilation and CPAP. While higher in the ST group, the number of infants requiring mechanical ventilation >24 h and CPAP >1 week, which could potentially impact feeding outcomes, were low and similar between groups (Table 1). Maternal characteristics were not different between groups, and all multiparous women had previously breastfed.

**Table 1:** Maternal and infant characteristics for the entire cohort as well as broken down by Calmita teat (CT) and standard teat (ST) groups, presented as mean ± standard deviation or percentage (count).

	Overall	CT Group	ST Group
**Maternal characteristics**	n = 51	n = 24	n = 27
Age (years)	32.2 ± 5.6	32.3 ± 5.6	32.2 ± 5.7
SEIFA percentile^+^	61.7 ± 27.1	61.2 ± 22.8	62.1 ±30.8
Multiparous %	51% (26)	54% (13)	48% (13)
Multiple birth	23.5% (12)	21% (5)	26% (7)
Intended BF duration (months)	11.8 ± 5.2	12.3 ± 4.6	11.5 ± 5.7
Rates BF “very important”	90% (46)	83% (20)	96% (26)
24 h milk production (mL)	726 ± 425	745 ± 290	709 ± 522
**Infant characteristics**	n = 60	n = 29	n = 31
Birth gestation (weeks)	30.7 ± 1.6	30.6 ± 1.6	30.8 ± 1.6
Birth weight (g)	1516 ± 346	1581 ± 340	1456 ± 345
Male	62% (37)	62% (18)	61% (19)
Small for gestational age	8.3% (5)	7% (2)	10% (3)
Mechanical ventilation	37% (22)	21% (6)	52% (16)
Mechanical ventilation >24 h	8.3% (5)	7% (2)	10% (3)
CPAP*	87% (52)	76% (22)	97% (30)
CPAP > 7 days	15% (9)	14% (4)	16% (5)
Late onset sepsis	8.3% (5)	10% (3)	6% (2)

+SEIFA percentile Socio-Economic Indexes For Areas ranks geographic area codes in Australia by relative socio-economic disadvantage

^ Fisher’s exact test

*CPAP: continuous positive airway pressure

All infants were offered daily breastfeeds from 33 weeks PMA. The first bottle feed was introduced at PMA 34.1 ± 0.6 for the CT group, which was 2 days earlier than for the ST group at PMA 34.4 ± 0.6 (HR = 2.13, 95% CI = 1.15–3.94, p = 0.02). CT infants commenced bottle feeds using the starter teat and changed to the advanced teat on completion of the first full oral feed, typically within one week of introduction of bottle feeds (0.89 ± 1.0 weeks).

The majority of infants completed their first full oral feed at a bottle (CT 83%; ST 86%; RR = 0.96, 95% CI = 0.76–1.23, p = 1.0) rather than at the breast. Timing of achievement of the first full oral feed was similar between groups (CT group 34.6±0.9 weeks PMA, ST group 34.8±1.2 weeks PMA, HR = 1.38, 95% CI = 0.77–2.47, p = 0.28). No other variables were significantly related to PMA at achievement of first full oral feed (Table 2).

**Table 2:** Univariate Cox proportional hazards results from analysing PMA at achievement of first full oral feed, and at achievement of full oral feeds over 24 h.

	PMA at achievement of first full oral feed			PMA at achievement of full oral feeds over 24 h		
Variable	HR	95% CI	P-Value	HR	95% CI	P-Value
**Group** CT vs. ST	1.38	0.77–2.47	0.28	1.25	0.28–5.58	0.77
**Singleton** Single vs. multiple birth	1.05	0.56–1.98	0.87	1.14	0.58–2.25	0.71
**Appropriate for gestational age** Yes vs. small	1.82	0.56–5.93	0.32	2.13	0.75–6.05	0.16
**Mechanical ventilation** ≤1 day vs. >1 day	2.41	0.58–10.01	0.23	3.89	0.91–16.57	0.07
**CPAP** ≤1 week vs. >1 week	1.20	0.47–3.06	0.70	1.74	0.73–4.19	0.22
**Birth gestation** For a 1 week increase	1.00	0.84–1.19	1.0	1.27	1.05–1.54	0.02*
**Maternal age** For a 5 year increase	0.90	0.69–1.19	0.46	1.04	0.78–1.39	0.79
**24 h milk production** For a 1 SD (425 mL/24 h) increase	0.92	0.62–1.36	0.67	1.01	0.78–1.31	0.94
**SEIFA percentile**^+^ For a 1 SD (27%) increase	1.11	0.80–1.53	0.53	1.11	0.83–1.48	0.47

*Significant multivariately

+SEIFA percentile Socio-Economic Indexes For Areas ranks geographic area codes in Australia by relative socio-economic disadvantage

Timing of achievement of 24 h of full oral feeds was similar between CT and ST groups (average PMA 36.7±1.2 vs. 36.7±0.9 weeks respectively; HR = 1.25, 95% CI = 0.28–5.58, p = 0.77). Infants born at a later gestation were significantly more likely to achieve full oral feeds at an earlier PMA than infants born at an earlier birth gestation (for a 1 week increase: HR = 1.27, 95% CI = 1.05–1.54, p = 0.015). No other variables were significantly related to timing of achievement of full oral feeds. (Table 2)

Infant weight at discharge was similar between groups (CT 2694±421g; ST 2586±423g; MD = 156g, 95% CI = -168-480g, p = 0.27) as was PMA (CT 37.2±1.2; ST 37.3±1.2; MD = -0.09, 95% CI = -0.8–0.62, p = 0.76). While the proportions of infants receiving breast milk at discharge were similar between groups (CT 100%, ST 90%; RR = 1.08, 95% CI = 0.97–1.20, p = 0.49), a significantly higher proportion of CT group infants were exclusively fed breast milk (CT 96%; ST 65%; RR = 1.36, 95% CI = 1.05–1.76, p = 0.028).

**Fig 2:**
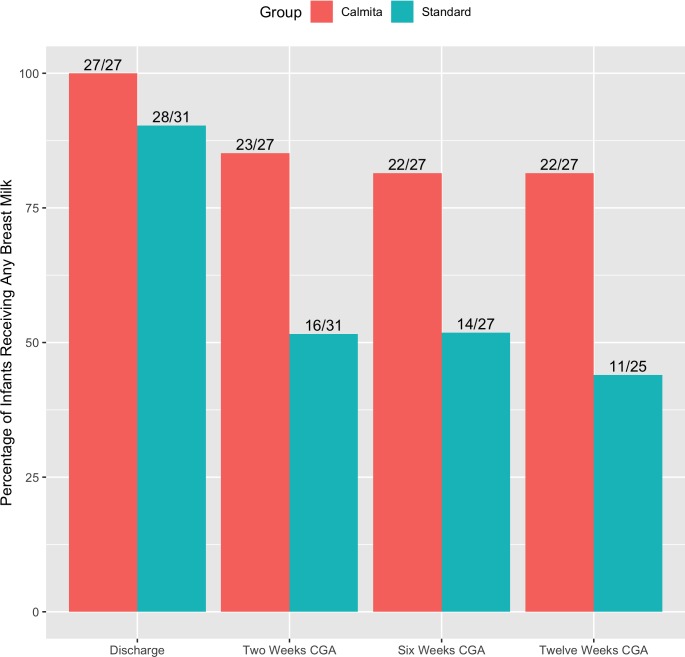
Percentages of any breast milk feeding at discharge, 2 weeks, 6 weeks and 3 months CGA.

When compared to the ST group, a higher proportion of the CT group breastfed and/or fed EBM up to 12 weeks corrected gestational age (CGA). Specifically, more CT infants were fed breast milk at 2 weeks (CT 85%; ST 52%; RR = 1.57, 95% CI = 1.08–2.27, p = 0.016), 6 weeks (CT 81%; ST 52%; RR = 1.42, 95% CI = 0.96–2.08, p = 0.069) and 12 weeks CGA (CT 81%, ST 44%; RR = 1.65, 95% CI = 1.05–2.61, p = 0.02; see Fig 2). One mother from each group was unable to breastfeed due to latch issues; they both exclusively fed EBM by bottle up to 6 weeks CGA before weaning or introducing some formula feeds.

**Fig 3:**
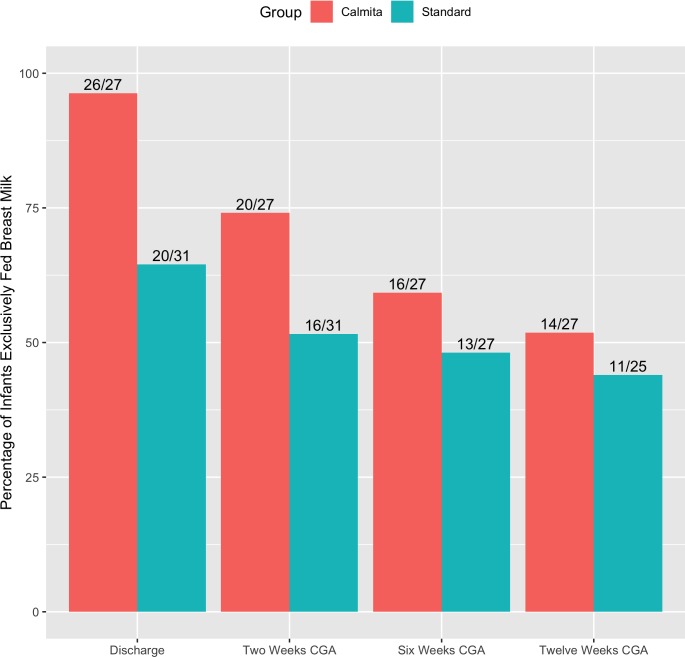
Percentages of exclusive breast milk feeding at discharge, 2 weeks, 6 weeks and 3 months CGA.

While more CT group dyads achieved exclusive breast milk feeding at 2 weeks CGA (CT 74%; ST 52%; RR = 1.41, 95% CI = 0.94–2.10, p = 0.091), 6 weeks CGA (CT 59%; ST 48%; RR = 1.12, 95% CI = 0.69–1.84, p = 0.64) and 12 weeks CGA (CT 52%; ST 44%; RR = 1.13, 95% CI = 0.65–1.96, p = 0.66, Fig 3) the differences were not statistically significant.

Limiting the dataset to mothers providing any breast milk, rates of exclusive breastfeeding (ie feeding directly at the breast only) were similar between groups at 2 weeks (CT 57%, ST 31%; RR = 1.89, 95% CI = 0.86–4.19, p = 0.08), 6 weeks (CT 50%; ST 57%; RR = 0.90, 95% CI = 0.50–1.64, p = 0.74) and 12 weeks CGA (CT 45%, ST 55%; 1.03, 95% CI = 0.52–2.06, p = 1.0). One set of twins in CT group achieved exclusive breastfeeding by 2 weeks CGA and continued to beyond 12 weeks CGA.

The CT group were provided with Calma teats at discharge to provide the option of continued use of a vacuum release teat during the transition to breastfeeding; they were not provided to the ST group, and were commercially available for purchase in the community. A small number in each group reported use of the Calma teat use after discharge ie. 2 weeks CGA; CT n = 4, ST n = 2; 6 weeks CGA; CT n = 5, ST n = 1 and 12 weeks CGA; CT n = 2, ST n = 1.

No adverse or unintended effects were noted for either study group.

## Discussion

For infants born <33 weeks gestation that were establishing breastfeeding, use of a vacuum release teat for bottle feeds when the mother was not available to breastfeed, was associated with significantly longer duration of breast milk feeding up to 12 weeks CGA, despite no differences between the vacuum release teat and conventional teat groups in terms of attainment of early feeding milestones. Further, infants in the vacuum release teat group were significantly less likely to be fed any infant formula at discharge.

The ultimate goal for the preterm infant-mother dyad is to achieve full breastfeeding however this is complicated by many factors. Early feeding milestone achievements are often interpreted as indicators of feeding maturity, yet feeding trajectories can differ widely between preterm infants born at similar gestational ages [5], suggesting inter-individual differences in the rate of feeding maturation. Commensurate with this theory we found that while the two groups reached early feeding milestones at the same time, after discharge home, infants in the vacuum release teat group were fed breast milk for significantly longer than their control group counterparts. Use of a vacuum release teat was uncommon after discharge home, and therefore not likely to have impacted feeding outcomes. Our findings suggest that use of the vacuum release teat during the neonatal unit stay may support the development of breastfeeding skills.

The study findings concur with that of a previous study that compared use of a prototype vacuum release teat and standard teat in preterm breastfed infants. The timing of achievement of early feeding milestones was similar between groups, higher proportions of infants in the vacuum release teat group were exclusively fed breast milk at discharge, and while not statistically significant, higher proportions of those infants received breast milk to 12 weeks post discharge [18].

Indeed preterm birth results in interruption of the pre-learning phase and disruption of the imprinting phase of oral feeding [10], with absence or deprivation of pre-learning activities such as infant-directed hand to mouth movements and sucking on digits, and swallowing and tasting of amniotic fluid [21]. Exposure to potentially noxious stimuli such as oral suction and hygiene practices and the presence of an intra-gastric feeding tube, and feeding methods that are disparate with breastfeeding may also disrupt the development of breastfeeding skills in the preterm infant. A vacuum release teat is designed to facilitate oro-motor actions that are similar to that required of breastfeeding [22] and therefore may protect to some degree the imprinting of normal tongue movement, application of intra-oral vacuum and control of flow in the context of feeding. In contrast, infants use both positive and negative pressure to remove milk from a standard teat, and milk may flow freely rather than in response to oral feeding movements [23].

Support of physiologically normal oro-motor feeding actions likely promotes the development of breastfeeding skills, enhancing the infant’s ability to transfer milk from the breast. As effective milk removal is essential to continued milk synthesis and the development of a full milk supply, it is possible that infants that have been exposed to a vacuum release teat achieve more effective suckling at the breast, which in turn promotes continued feeding of breast milk, whether by direct breastfeeding or in combination with feeding of EBM.

Our study findings are clinically important, as breast milk optimises the health and development of preterm infants, both in the short and longer term. Longer duration and ‘dose’ or exclusivity of breast milk feeding imparts greater benefits [3, 24]. It is imperative that the perspective of the mother is considered with regard to preterm infant feeding following discharge. For mothers that intend to breastfeed, achievement of direct breastfeeding is described as an empowering experience that contributes to bonding and maternal role satisfaction [25, 26]. When faced with breastfeeding difficulties after discharge, mothers of infants born preterm may choose to feed EBM. The workload associated with expressing, storing and feeding breast milk may be considered demanding or unsustainable [25]. It is therefore logical that breastfeeding, rather than breast milk feeding, at the time of hospital discharge is associated with longer breastfeeding duration [14] [27]. While use of a vacuum release teat during the neonatal unit stay may not provide immediate measurable benefits such as earlier achievement of oral feeding, it likely contributes to longer term health and developmental advantages to the infant and role satisfaction for the mother through its significant effect on breastfeeding duration.

Findings from this study are limited to healthy infants born < 33 weeks gestation, and to mothers with a high motivation to breastfeed and milk production >300 mL/24 h. It was not possible to blind the intervention from the researchers, mothers or nurses caring for the infants.

## Conclusion

Wherever possible, mothers of hospitalized preterm infants that intend to breastfeed should be supported with regular opportunities for breastfeeding. When the mother is not available to breastfeed, use of a vacuum release teat for oral feeds may offer an effective strategy for promoting development of oro-motor feeding skills which impact continuation of breastfeeding beyond discharge.

## Supporting information

S1 File CONSORT checklist. (PDF)

S2 File Study protocol. (PDF)
