# Supplementary material for: Effect of vacuum–release teat versus standard teat use on feeding milestones and breastfeeding outcomes in very preterm infants: A randomized controlled trial
Source: PLoS One. 2019 Mar 22;14(3):e0214091. doi: 10.1371/journal.pone.0214091 (PMC6430377; doi:10.1371/journal.pone.0214091)
Supplement: S2 File — (PDF) [file pone.0214091.s002.pdf]

Development of Breastfeeding Skills in Preterm Infants

Trial start date: February 2015 **Trial finish date:** February 2016

Coordinating Principal Investigator: W/Prof Karen Simmer
Investigators A/Prof Donna Geddes, Sharon Perrella RN RM IBCLC MSc pending PhD
Associate Investigators: Yen Kok RN,RM,NNT,MNsg,cHPEd, Judy Adams RN RM IBCLC, Sandra Andersen RN RM IBCLC

Trial Summary

Background: Whilst breastfeeding is the desired feeding outcome for preterm infants, little is known about the development of preterm breastfeeding skills and the impact of bottle-feeding teats, nipple shields and degree of breast fullness on the achievement of full oral feeding. At King Edward Memorial Hospital (KEMH), there are limited rooming-in facilities for mothers of preterm and sick infants who may remain inpatients for up to 16 weeks. Mothers try to visit daily but distance, family and work commitments may limit this and mothers are rarely able to be in the neonatal intensive care unit (NICU) for all of their infant's suck feeds. Bottle feeds are required occasionally. There is evidence that an intraoral vacuum-triggered teat (Calmita, Medela AG) encourages a sucking action similar to breastfeeding and reduces the length of hospital stay and formula use at discharge compared to a conventional teat. We wish to examine the effects of different teats and breastfeeding support on feeding outcomes.

Objectives:

1. To track maturation of intraoral vacuum, SSB coordination and physiological stability over time and determine if this is related to increased breastfeeding effectiveness and efficiency.
2. To explore the effect of breast fullness on milk transfer at 34 weeks corrected gestational age (CGA).
3. To explore the use of a nipple shield on milk transfer at 35 weeks CGA.
4. To compare SSB coordination and maturation during breastfeeding with that during bottle feeding with Calmita, and bottle-feeding with a conventional teat.
5. To determine the effect of a vacuum triggered teat on timing of achievement of full breastfeeding and on breastfeeding duration

Trial Plan:

A randomised controlled trial of 60 preterm infants will be carried out. When an infant is ready for a suck feed but mother is unable to be present, bottle feeds will be given using a conventional teat (control group) or Calmita teat (intervention group). Weekly breastfeeds and bottle-feeds will be monitored for SSB coordination, heart rate, oxygen saturation and milk intake. Infants will have a single breastfeed monitored at 33 weeks CGA, and up to 2 breastfeeds monitored over 2 consecutive days at 34 weeks CGA (breastfeeds from 'full' and 'empty' breasts) and at 35 weeks CGA (with and without a nipple shield). The intervention group will be given the Calma teat (term version of Calmita) at discharge from the study hospital.

Breastfeeding outcomes will be tracked by follow up phone calls up to 3 months post term.

Background

Whilst breastfeeding is the ultimate feeding outcome, breastfeeding duration is significantly shorter for infants born < 34 weeks gestation [1]. Little is known about the development of breastfeeding skills in hospitalised preterm infants. Further while achievement of full oral feeding is usually a requirement for discharge from hospital, the impact of different bottle-feeding teats on its timing is not clear. It is assumed that the removal of small milk volumes from the breast is due to low intraoral vacuums and immature suck-swallow-breathe (SSB) coordination however the influence of factors such as fullness of the breast, and the use of nipple shields, and different bottle-feeding teats have not been well investigated.

Maturation of preterm infant SSB coordination during breastfeeding

Suck-swallow-breathe coordination is one of the most complex tasks required for safe and efficient feeding. Preterm infants often lack the maturity required for effective coordination and this is a major impediment to swift progression of oral feeding. No studies have precisely described the temporal relationship of SSB coordination nor have they investigated maturation of SSB coordination during the establishment of breastfeeding. Some data exists for bottle-feeding however direct translation is impossible due to the various study teats used, and differences in milk delivery with dissimilar oro-motor movements between breast and bottle-feeding. A study of bottle-fed preterm infants tracked from 34 though to 42 weeks CGA showed that effective SSB coordination is reliant on two factors; a consistent suck-swallow ratio of 1:1 or 2:1 and timing of the swallow during a safe point in the respiratory cycle ie. at the beginning of either inspiration or expiration [2]. Further significant maturation of feeding behaviour has been observed between 33 and 36 weeks CGA in a cohort of infants born at 28 to 31 weeks gestation[3]. Nipple shields may be employed to facilitate the preterm infant's attachment to the breast and improve milk transfer. This practice is based on the work of Meier et al.[4], however there are no standard guidelines for the implementation and transitioning from nipple shield use. Given that full breastfeeding would be the best possible outcome for these infants in terms of both short and long-term health very little basic research exists as a foundation on which to build effective clinical protocols.

Effect of delivery of milk via different teats on SSB co-ordination in preterm infants

For preterm infants who have demonstrated the ability to attach and suck at the breast, in the absence of the breastfeeding mother it is common for some intra-gastric tube feeds to be replaced with bottle feeds with the rationale that more frequent suck feeds accelerate the progression to full oral feeding. This translates to the situation where it is rare for an infant not to receive a bottle during their hospital stay. One of the problems associated with bottle-feeding using conventional teats is the occurrence of apnoea, which is more frequent than with breastfeeding. Most recently we have shown that when hospitalised preterm infants are fed with a teat that does not release milk unless the infant creates a vacuum (Calmita, Medela AG, Switzerland), a sucking action similar to breastfeeding is facilitated, and this is

associated with improved breastfeeding rates at discharge and decreased length of hospital stay when compared to infants fed with a conventional teat. The incidence of any breastfeeding at discharge was similar between the groups (*Simmer et al, Novel feeding system to promote establishment of breastfeeds after preterm birth: a randomised controlled trial. Submitted*). It is possible that use of the Calmita teat facilitates coordination of SSB by the infant's regulation of milk flow. If so one would expect a reduction in apnoea and bradycardia during feeding with Calmita compared to the conventional teat. Further after discharge many preterm infants continue to require top up feeds from a bottle while transitioning to full breastfeeding. As the Calmita teat was not available following discharge from hospital it was not possible to determine whether continued use a vacuum controlled teat such as the Calmita or Calma (Medela AG, Switzerland) for top up feeds post discharge influences duration of breastfeeding and achievement of full breastfeeding

Trial Aims

1. To track maturation of intraoral vacuum and SSB coordination as well as physiological stability of breastfeeding preterm infants over time and determine if this is related to increased effectiveness and efficiency of breastfeeding. We hypothesize that improvement in breastfeeding efficiency (mL/min) and effectiveness (total mL transferred) will be associated with changes in one or more of vacuum strength (average and changes across a feed), consistency of sucking (number of sucks/suck bursts per feed) and SSB coordination.
2. To explore the effect of the volume of milk in the breast on milk transfer during breastfeeding at 34 weeks CGA. Our hypothesis is that more milk in the breast will result in a higher volume of milk being transferred by the infant at the breastfeed.
3. (Optional) To explore the effect of nipple shield use on milk transfer at 35 weeks CGA. The hypothesis is that nipple shields improve attachment and breastmilk transfer
2. To compare SSB coordination and maturation during breastfeeding with bottle feeding with a Calmita teat, and bottle-feeding with a conventional teat. Our hypothesis is that infants fed with a Calmita teat will experience less apnoea and bradycardia and reach full oral feeds more rapidly than those fed with a standard teat.
3. To determine the effect of a vacuum triggered teat (Calmita during stay in study hospital, and Calma used for top up feeds post discharge/transfer from the study hospital) on the timing of achievement of full breastfeeding and on breastfeeding duration.

Trial Design

Trial endpoints

The primary endpoint for this trial is total breastfeed volume (mL) transferred at a breastfeed, measured at 33, 34, 35 weeks and term CGA

Secondary endpoints to be measured during monitored feeds are

efficiency (mL/min)

- total suck duration

- total suck bursts
- mean, peak and baseline intraoral vacuum (mmHg)
- oxygen saturation range
- heart rate range
- incidence of bradycardia
- incidence of oxygen desaturation

The following feeding endpoints will also be determined

- timing of achievement of full oral feeds
- timing of achievement of full breastfeeding
- breastfeeding duration (monitored up to 3 months post-term)

1. A randomized controlled clinical trial will be undertaken with random allocation in blocks of size 4 to either a control group or an intervention group. Study design is depicted in Figure 1.
2. A pilot study of up to 5 infants will be completed prior to commencement of the trial to test and refine the set-up and duration of monitoring sessions.
3. Study participant data and milk samples will be re-identifiable, with an identification code assigned to each infant. A master sheet that matches infant identity to identification codes will be stored in a password-protected electronic file on a lap top computer. All paper copies of case report forms will be stored in a locked filing cabinet at The University of Western Australia.
4. The effect of a vacuum-controlled bottle-feeding teat (Calmita and Calma teats, Medela AG) on breastfeeding outcomes will be evaluated in this clinical trial. A shut-off valve is incorporated into the Calmita teat so milk flows only when the infant creates a vacuum. Venting prevents collapse of the teat. There are two versions of Calmita with different threshold levels for the vacuum-controlled valve of -10±5 mmHg (starter teat) and -30±15 mmHg (advanced teat). We have previously investigated a prototype of this product but wish to carry out more extensive studies on the final product.

   Summary of findings from previous studies

   A randomised controlled trial of 97 preterm infants (birth gestation; Intervention: $30^1 \pm 2^4$; Control: $30^1 \pm 2^5$ weeks) was carried out at Special Care Nurseries (SCN) KEMH from August 1st to June 30th 2012 to compare Calmita with the conventional teat in use in the neonatal unit. Primary outcomes showed that time to full suck feeds were not different between the Control group and Intervention group (Calmita; p=0.24). The Intervention group had shorter length of stay in hospital with a mean difference of 2.5 days for CGA at discharge (p=0.026) and were discharged lighter. The Intervention group also received less formula at discharge (Intervention: 35%; Control: 16%, p=0.036), but did not differ with respect to breastfeeding rates. It was concluded that use of Calmita during establishment of breastfeeding reduced duration of hospitalisation of preterm infants.

   Simmer K, Kok C, Nancarrow K, Hepworth AR, Geddes DT. Novel feeding system to promote establishment of breastfeeds after preterm birth: a randomised controlled trial. Journal of Paediatric and Child Health (under review).

Criteria for termination of the trial:
Individual participants. Infants that demonstrate physiological instability ie. oxygen desaturations / bradycardia that require intervention (supplemental oxygen, or bag and mask ventilation) during a monitored study feed, where there have been no other episodes in the previous 24 hours, will be referred for review by clinical staff and advice sought re whether the infant should be withdrawn from the trial.
Entire trial: Safety of the trial will be monitored continuously and formally reviewed at interim analysis ie. at recruitment of 15 infants to each arm of the study to determine whether any adverse events have been reported as a result of monitoring or use of the trial teat. In that case, the trial will be stopped while the events are fully investigated, reported to WNHS Ethics Committee, and continuation of the trial re-considered.
We do not anticipate any adverse effects of the vacuum-triggered trial teat as data from our previous study indicates no change in physiological stability during its use of whereby, unlike a conventional teat, milk flow is controlled by the infant during feeding.

5. The expected duration of the trial is 12 months. The total duration of participation of mothers and infants is up to 43 weeks including intermittent monitoring over 3 weeks during hospitalization, one study site visit (likely to coincide with a scheduled hospital follow-up visit) and up to 3 follow up phone calls following discharge from the study site.

Table 1: Schedule of Assessments

**Monitoring during SCN stay**	Corrected gestational age (weeks)			
	33	34	35	36+
Breastfeed (usual condition for each baby)	✓		✓	Invited
Breastfeed: full breast		✓		
Breastfeed: empty breast		✓		
Breastfeed: nipple shield				
Breastfeed: no nipple shield			Invited if shield normally used	
Bottlefeed: allocated conventional / Calmita teat	✓	✓	✓	Invited
Maternal 24hr milk production study	✓	✓	✓	
Pre and post weighs with every breastfeed	Throughout SCN stay			
Oral feeds with allocated teat where mother not available to breastfeed	Throughout SCN stay			
**Monitoring post discharge**	Corrected postnatal age (weeks)			
	term	2	6	12
Breastfeed (usual condition for each baby)	✓			
Follow up phone call		✓	✓	✓

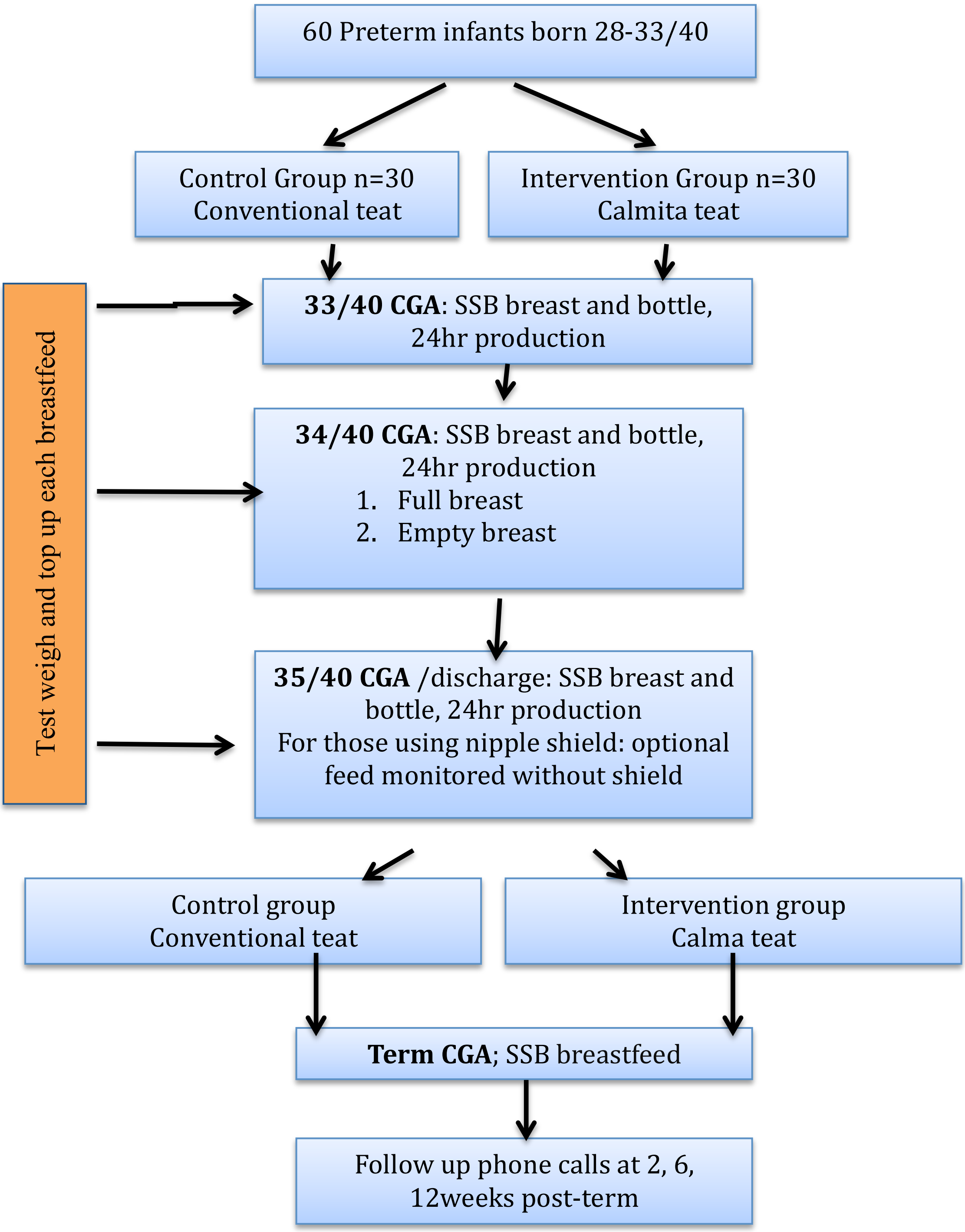

Figure 1. Study Design

Source and Selection of Participants

Participants will be recruited from the Special Care Nurseries at KEMH. We will recruit 60 mothers and their healthy preterm infants born between 28-33 weeks gestation with a home address within a 35km radius of the hospital.

Inclusion criteria will include medically stable infants, and mothers with an intention to breastfeed and total daily breastmilk expression volume of at least 300 mL/24hr.

Exclusion criteria will include mothers with a history of breast surgery, infants receiving CPAP or mechanical ventilation at 34 weeks CGA, acute illness and/or infection and congenital disease or malformation.

Treatment of Participants

1. When the mother is not available to breastfeed, control group infants will use a conventional teat for suck feeds whereas the interventional group infants will use the Calmita teat. The starter teat will be offered initially and when the infant takes full feeds from the starter teat for 48-72 hours they will then use the advanced teat.
2. Monitoring of a weekly breastfeeds and bottle feeds with the allocated teat will be performed at 33, 34 and 35 weeks CGA and involves the following
   - Weigh infant pre and post breastfeeding
   - Intraoral ultrasound
   - Continuous Intraoral vacuum measurements
   - Respiratory Inductive plethysmography
   - Continuous pulse oximetry for oxygen saturation (SaO_2) and heart rate
   - (optional) Maternal pre/post breastfeed milk samples <1.0mL
   - Maternal rating of Preterm Infant Breastfeeding Behavior Scale[5] (PIBBS, Appendix A)

   SSB coordination is evaluated using data from intraoral vacuum, respiration and swallowing (Respiratory Inductive Plethysmography) heart rate and oxygen saturation obtained throughout the entire breastfeed[6].
3. Test weighs of the infant pre and post feed, for all feeds, will be made to determine milk intake and allow calculation of top up feeds. For monitored feeds, pre and post feed milk samples (<1.0mL) will be collected and fat content measured to provide a further estimate of breast fullness.
4. At each monitored breastfeed the Preterm Infant Breastfeeding Behavior Scale[5] (PIBBS, Appendix A) will be completed by the mother.
5. In the study setting is it assumed that most stable preterm infants are offered (at least daily) breastfeeding experiences from 32 weeks CGA. A weekly breastfeed with be monitored from 33 weeks CGA. At 34 weeks CGA each infant will have up to 2 breastfeeds and one bottle-feed monitored in random order on 2-3 consecutive days with a full or empty breast. A full breast is one that has not been expressed for 3 hours prior to feeding and an empty breast one that has been expressed within an hour of the feed. At 35 weeks CGA those using a nipple shield will have the option of also having a monitored breastfeed without a nipple shield.
6. At 35 weeks or at discharge/transfer each infant will have 1 breastfeed monitored with or without a nipple shield depending on whether the infant is typically feeding with a shield, and one bottle-feed will be monitored. Infants

that remain in the Neonatal Nursery beyond 35 weeks CGA will be invited to continue with weekly monitored breastfeeds and bottle feeds until the time of transfer or discharge.

7. Each week the mother will be given the option of collecting a small milk sample (0.5-1.0mL) before and after every pumping or breastfeed over a 24-hour period to allow more accurate estimation of breast fullness. We expect milk production to increase over the study period therefore one 24-hour collection will not be sufficient.
8. Prior to discharge each mother will be asked to record expression volumes and the milk transfer at the breast (test weigh as in point 5) for a 24-hour period. This will be compared to the normal term infant ranges for 24 hour milk production/milk transfer.
9. At discharge/transfer the mother in the intervention group will receive Calma (term) teats to use for top up feeds while establishing breastfeeding.
10. The mother and infant will be invited to return to KEMH at term corrected age for monitoring of SSB during a breastfeed

The researchers will monitor compliance with use of the allocated teats and pre-post feed weighing during participants' hospital stay, and will be responsible for providing Calma teats to the intervention group mothers prior to the infants' transfer or discharge from KEMH.

Data collection techniques, participant tasks and time involved

Monitoring of weekly daytime breastfeeds will not require extra time for the mother or infant and will last from 15 to 60 minutes depending upon the feed duration. The following data collection techniques are used:

Intra-oral vacuum

Intra-oral vacuum (relative to atmospheric, mmHg) will be measured via a small Silastic tube (SNS, Medela AG, Baar, Switzerland) attached to a pressure transducer (Cobe Laboratories, Frenchs Forest, NSW, Australia) taped alongside and terminating just past the nipple tip (2mm). The pressure transducer will be connected to a bridge amp (AD Instruments, Castle Hill, NSW, Australia) via an interconnect cable (Cobe Laboratories). The output of the pressure transducer and ultrasound images will be channeled to a Power Lab (ADInstruments, Castle Hill, NSW, Australia) and simultaneous recordings made using the software package Chart v4.5 (AD Instruments) on a laptop computer. The Silastic tube is very fine and soft; very young infants do not appear to be aware of the presence of the tube and its presence does not cause any discomfort to the breastfeeding mother.

Ultrasound Imaging

Submental mid-sagittal scans of the infant's oral cavity will be performed for the duration of the entire feed. The infants will be scanned using a Sonologic SonoScape (Brisbane, Australia) with a 5PI phased array transducer and Parker Ultrasonic Gel (Fairfield, New Jersey, USA). Two focal zones will be employed to narrow the ultrasound beam and improve image resolution. One will be located at the level of the hard palate and the other at the nipple–tongue border. All scans will be channeled into the Power Lab and recorded for later analysis.

Respiration and swallowing measurements

Patterns of infant respiration and swallowing will be measured using respiratory inductive plethysmography (RIP), (Respitrace QDC, SensorMedics, Yorba Linda, CA, USA) with two bands; one placed around the thorax at the level of the nipples and a second around the abdomen at the level of the umbilicus. Bands will be secured using Micropore tape if necessary. The bands are connected to the Respitrace and channeled into the Power Lab. RIP has been validated against ultrasound as a highly reliable method for identifying swallows during breastfeeding [7] and has been used successfully to compare respiratory changes for breast and bottle-feeding in term infants [8].

Oxygen saturation and heart rate monitoring

Oxygen saturation and heart rate monitoring will be performed for the duration of the test feeds. (As per SCN policy all infants <34/40 are continuously monitored). For infants no longer requiring monitoring, pulse oximetry will be used for monitored feeds, with a sensor attached to the infants' foot. The research nurse will record any episodes of oxygen desaturation and bradycardia during the feed. These events will be documented on the intraoral vacuum and respiratory traces to enable retrospective identification of the episodes.

Infant milk intake

To determine milk intake infant test weighs will be performed for all breastfeeds. Electronic scales (BabyWeigh Scale, Medela AG, Baar, Switzerland, sensitive to 2 grams) will be used to weigh infants before and immediately after the feed. The amount of milk consumed is calculated as the difference in grams between the pre-feed and post-feed infant weights, and 1gram ≈ 1mL. Milk transfer (g/minute) is calculated as the amount of milk consumed during the feed (g) divided by the duration of the feed (minutes). Knowledge of milk intake during a breastfeed will allow for accurate calculation of top up feeds.

Estimation of Degree of Breast Fullness

Breastmilk fat concentrations increase as the volume of milk available within the breast reduces. Changes in breastmilk fat concentration over the course of a breastfeed or expression provide an indication of fullness, and when pre/post samples are collected over a 24hr period, estimation of the degree of fullness can be made [9, 10]. A small milk sample (<1.0mL) will be taken before and after monitored feeds, and mothers will have the option of collecting pre/post samples when recording their 24hr milk production.

Preterm Infant Breastfeeding Behavior Scale[5] (PIBBS, Appendix A)

The PIBBS rates aspects of breastfeeding such as rooting, attachment, sucking and swallowing using a six item scale, with higher scores indicating greater competence. The scale has a reported inter-rater reliability of 0.68 to 0.94. Completion of the PIBBS takes <5 minutes. Mothers will be asked to complete the PIBSS after each monitored breastfeed.

Assessment of Efficacy

Efficacy of the trial teat will be evaluated through measurement of the primary endpoint ie. total volume transferred at the breast, and secondary endpoints that relate to breastfeeding outcomes (eg. volume transferred per minute, range and average intraoral vacuum, achievement of full oral feeds, full breastfeeding and breastfeeding duration) and safety (eg. SSB coordination and incidence of apnoea and bradycardia during feeding). To assess efficacy, total volume transferred at the breast will be examined at interim analysis when complete data is available for 15 infants in each group, and again on completion of the study.

Assessment of Safety

There are no foreseeable risks to the infant or mother. We do not anticipate any adverse effects of the vacuum-triggered trial teat as data from our previous study indicates no change in physiological stability during its use whereby, unlike a conventional teat, milk flow is controlled during feeding. To ensure safety, the incidence of apnoea and bradycardia, and any other adverse effects, will be recorded at each feed and examined at interim analysis when complete data is available for 15 infants in each group.

An expected benefit of study participation is better infant growth (in both infants fed with the conventional teat and those fed with Calmita) because breastmilk transfer will be measured for every breastfeed and top up feeds calculated to meet the volume prescribed by the neonatologist. Currently top up feed volumes are estimated based on assumptions about the volume taken at the breast – data from our previous studies indicate that the assumed volume is frequently overestimated.

Mothers in this study may also be encouraged to continue breastfeeding simply due to the monitoring and follow up over the course of the study, whereby breastfeeding duration is tracked; this may result in longer breastfeeding duration.

Statistical analysis, data management, and record keeping

The primary outcome measure for this study is the total volume transferred during a breastfeed. Meier reported average transfer volumes of 18.4mL±13.2 and 3.9mL±7.0 with and without nipple shield, respectively [11]. Samples sizes required to detect a clinically relevant effect of 5mL difference between feeds with full and empty breasts were calculated separately for these two conditions. The more conservative calculation is that the study would need 24 infants in each treatment group to have 80% power (Type I error rate α=0.05) of detecting a difference this large. Allowing for a possible dropout rate of 20%, as was observed in a similar study, 30 infants per group will need to be recruited at 33 weeks CGA. The sample size was calculated as if the data will be analysed using an independent samples *t*-test. Actual analyses will be more powerful, and thus will have power to detect smaller differences.

Using this sample size, an independent samples *t* test would have the power to detect differences in secondary outcomes equivalent to 0.83 * SD. This is equivalent to differences of approximately 3.9 min total sucking duration, and 12 suck bursts per feed. As with the primary outcome, use of covariate data, repeated measurements and more powerful analytic techniques increase the likelihood of detecting smaller differences than those determined here.

Data will be analysed on an intention to treat basis using R 2.15 or later versions (The R Core Team)[12] with nlme,[13] lattice,[14] and multcomp[15] packages for linear mixed models, graphical exploration and general linear hypothesis tests, respectively. To monitor both safety and efficacy, interim analysis will be performed when complete data is available for 15 infants in each group, with p values set to p=0.029 for both interim and final analyses as per Pocock (p372, [16]).

For the monitored breastfeeds, differences in milk transfer volume as well as measured sucking, physiological, and feeding variables, linear mixed effects modelling will be used, with grouping for the random effects at the participant level. Use of nipple shield (yes/no), breast fullness (full/empty) and relevant demographic variables will be considered as predictive factors.

Paper records of data will be stored in a locked filing cabinet within a locked room at the Neonatology Clinical Care Unit (NCCU). Electronic data records will be backed up and both copies stored in password-protected files on a lap top computer.

Study investigators will transport re-identifiable milk samples from KEMH to the laboratories of the School of Chemistry and Biochemistry at The University of Western Australia where they will be secured in frozen storage at ≤ -20°C until the time of analysis.

Access to paper records and electronically stored data will be strictly limited to the study investigators. They will be securely stored for 15 years after publication of findings, after which time paper records will be shredded and electronic data will be overwritten.

Monitoring / Audit

The trial investigators will permit trial-related monitoring, audits and/or regulatory inspections, providing direct access to source data and documents as required by the WNHS Ethics committee.

Quality control and quality assurance

The trial will be conducted in compliance with Good Clinical Practice, as outlined in The Australian Clinical Trial Handbook (2006). Quality assurance has been addressed through consultation with neonatologists and lactation consultants at the study setting. Review of the trial protocol is to be completed by the Scientific Advisory Committee, Governance and Ethics Committees at KEMH.

Ethics

The research nurse (Kath Nancarrow, neonatal nurse) or principal research associate (Dr Sharon Perrella, neonatally trained nurse and lactation consultant) will approach mothers of preterm infants that meet the trial criteria. The mothers will be provided with a verbal explanation of the study and will be requested to read the provided written study information sheet. The nurse or associate will offer to return later to find out whether the mother would like to participate with her infant. Any requested additional information will be provided verbally and written consent obtained. (see Appendices B, C for Study Information and Consent forms). The lactation consultants employed by NCCU have been involved in the study design and will support the research nurse, research associate and patients when appropriate.

It is possible that the use of a teat that facilitates the sucking dynamics of a breastfeed may maintain physiological stability as well as provide 'training' that may advance the development of breastfeeding skills, shorten the transition to full breastfeeding, and increase breastfeeding duration. The knowledge gained from the study has the potential to form the basis for evidence-based modification of feeding policies for preterm infants. When initiating breastfeeding for very preterm infants, current advice often includes use a nipple shield to aid attachment, and breast expression prior to offering a breastfeed. There is no/little evidence for this advice. Evaluation of maturation of SSB coordination may also provide markers such as strength of vacuum that are predictive of breastfeeding success. In this case simple monitoring may provide a means for determining readiness for the transition to full breastfeeding.

The coordinating principal investigator (CPI) of the study is also a consultant neonatologist and the Director of the Neonatology Clinical Care Unit (study site). On occasion the CPI may be the assigned neonatologist responsible for the medical care of study participants. The CPI will not be directly involved in recruitment of participants or in data collection. All potential participants will be verbally encouraged to decline or consent freely and voluntarily with assurances that their decision will not impact in any way on their infant's present or future care.

Budget, financing, indemnity and insurance

The CPI is employed and insured by KEMH, while A/Prof Donna Geddes and Sharon Perrella are employed and insured by The University of Western Australia. Sharon Perrella's salary is partly funded by a Telethon research grant ($40,539) that is administered by Women's and Infants' Research foundation, and the Centre for Neonatal Research and Education has provided a donation of $53,000.

Teats for the intervention group will be donated by the manufacturer, Medela AG. Each infant (n=60) will require 30 starter and 60 advanced Calmita and 5 Calma teats.

Employment of a research nurse will be funded by the Centre for Neonatal Research and Education, The University of Western Australia.

Annual Rate ANF 1.8	$76,846.00
Weeks per annum	52.14
Weekly Salary	$1,473.84
Hours per week	40
Hourly Rate	$36.85
<u>On-Costs</u>	
Workers Comp 3.19%	$2,451.39
Superannuation 9.25%	$7,108.26
Long Service Leave 2.50%	$1,921.15
Annual Leave 7.69%	$5,909.46
Sick Leave 3.85%	$2,958.57
Processing costs 3.77%	$2,897.09
Full Year 1 FTE 30.25%	$100,091.92
Full Year at 0.5 FTE	$ 50,045.96
With GST	$ 55,050.56

The following research support will be given in kind
Sharon Perrella Level A Step 08 1.0 FTE = $85,942 – Telethon funding = $45403
A/Prof Donna Geddes Level B Step 02 @ 1 day / week = $22,700
W/Prof Karen Simmer Level E Step 01 @ 1 day / month = $7980
Choi Heen (Yen) Kok @ 1 day / fortnight = $10,720

Publication

The trial will be registered with the publicly accessible Australian New Zealand Clinical Trials Registry following confirmation of Ethics approval and prior to commencement of the trial. Within 6 months of the completion of data analysis, study findings will be disseminated through oral presentations, written reports, and manuscripts will be submitted to peer-reviewed professional journals for publication.
Study participants that request to be informed of study results will be provided with a plain language report and/or copies of published papers as requested.

References

1. Perrella, S.L., et al., *Influences on breastfeeding outcomes for healthy term and preterm/sick infants.* Breastfeed Med, 2012. **7**: p. 255-61.
2. Lau, C., *Oral feeding in the preterm infant.* Neoreviews, 2006. **7**: p. e19-e27.
3. Mizuno, K. and A. Ueda, *The maturation and coordination of sucking, swallowing, and respiration in preterm infants.* Journal of Pediatrics, 2003. **142**(1): p. 36-40.
4. Meier, P.P., et al., *Nipple shields for preterm infants: effect on milk transfer and duration of breastfeeding.* J Hum Lact, 2000. **16**: p. 106-14.
5. Nyqvist, K.H., et al., *Development of the Preterm Infant Breastfeeding Behavior Scale (PIBBS): a study of nurse-mother agreement.* Journal of Human Lactation 1996. **12**: p. 207-19.
6. Sakalidis, V.S., et al., *Oxygen Saturation and Suck-Swallow-Breathe Coordination of Term Infants during Breastfeeding and Feeding from a Teat Releasing Milk Only with Vacuum.* International Journal of Pediatrics, 2012. **2012**: p. 130769.
7. Geddes, D.T., et al., *Ultrasound imaging of infant swallowing during breast-feeding.* Dysphagia, 2010. **25**(3): p. 183-91.
8. Mathew, O.P. and J. Bhatia, *Sucking and breathing patterns during breast- and bottle-feeding in term neonates. Effects of nutrient delivery and composition.* Am J Dis Child, 1989. **143**(5): p. 588-92.
9. Kavanaugh, K., P.P. Meier, and J.L. Engstrom, *Reliability of weighing procedures for preterm infants.* Nurs Res, 1989. **38**(3): p. 178-9.
10. Kent, J.C., et al., *Volume and frequency of breastfeedings and fat content of breast milk throughout the day.* Pediatrics, 2006. **117**(3): p. e387-95.
11. Meier, P.P., et al., *Nipple shields for preterm infants: effect on milk transfer and duration of breastfeeding.* J Hum Lact, 2000. **16**(2): p. 106-14; quiz 129-31.
12. Team, T.R.C., *R: A language and environment for statistical computing*, in *Foundation for Statistical Computing*2008: Vienna, Austria.
13. Pinheiro, J., et al., *nlme: Linear and Nonlinear Mixed Effects Models*, in *R package* 2008.
14. Sarkar, D., *lattice: Lattice Graphics*, in *R package* 2008
15. Hothorn, T., F. Bretz, and P. Westfall, *Simultaneous inference in general parametric models.* Biomed, 2008. **50**: p. 346-363.
16. Piantadosi, S., *Clinical trials: a methodological perspective*. Wiley Series in Probability and Statistics, ed. W.A. Shewhart and S.S. Wilks2005, Hoboken: John Wiley & Sons, Inc.

Appendix A: Preterm Infant Breastfeeding Behavior Scale (PIBBS)*

Scale items	Maturational steps	Score
Rooting	Did not root	0
	Showed some rooting behavior	1
	Showed obvious rooting behavior	2
Areolar grasp (how much of the breast was inside the baby's mouth)	None, the mouth only touched the nipple	0
	Part of the nipple	1
	The whole nipple, not the areola	2
	The nipple and some of the areola	3
Latched on and fixed to the breast	Did not latch on at all so the mother felt it	0
	Latched on for ≤ 5 min	1
	Latched on for 6-10 min	2
	Latched on for ≥ 11-15 min	3
Sucking	No sucking or licking	0
	Licking and tasting, but no sucking	1
	Single sucks, occasional short sucking bursts (2-9 sucks)	2
	Repeated short sucking bursts, occasional long bursts (≥10 sucks)	3
	Repeated (≥ 2) long sucking bursts	
Longest sucking burst	1 – 5 consecutive sucks	1
	6 – 10 consecutive sucks	2
	11 – 15 consecutive sucks	3
	16 – 20 consecutive sucks	4
	21 – 25 consecutive sucks	5
	≥ 26-30 consecutive sucks	6
Swallowing	Swallowing was not noticed	0
	Occasional swallowing was seen	1
	Repeated swallowing was noticed	2

*Nyqvist et al, 1996

Appendix B: Participant Information Sheet

Development of Breastfeeding Skills in Preterm Infants

PARTICIPANT INFORMATION SHEET

Why are we doing the study?

It takes time for preterm babies to learn how to breastfeed, and the achievement of full oral feeds is required before infants can be discharged home. This research aims to provide evidence to support mothers of preterm infants establish breastfeeding. When mothers are not available to breastfeed their baby in hospital, bottle feeds are often given to encourage oral feeding. Use of a new teat that only releases milk when the baby sucks to create a vacuum in the back of his mouth on the teat (Calmita, Medela AG), has been shown to improve breastfeeding rates at discharge.

Milk flows readily from a conventional teat and flow increases with suck movements without need to develop vacuum. While it is known that the baby's coordination of sucking, swallowing and breathing, use of a nipple shield, different bottle-feeding teats and fullness of the mother's breast can impact breastfeeding, it is not well understood how each of these affect breastfeeding success.

The aims of this study are to

- examine the development of the baby's sucking strength together with coordination of swallowing and breathing at 33, 34 and 35 weeks corrected age (CGA) and again at term.
- examine the effect of the mother's breast fullness on the amount of milk that the baby takes from the breast at 34 weeks CGA.
- (Optional) examine the baby's sucking strength together with coordination of swallowing and breathing when feeding both with and without a nipple shield at 35 weeks CGA.
- compare the coordination of sucking, swallowing and breathing during breastfeeding with that during bottle feeding using a conventional teat (Control group) or a Calmita teat (Intervention group) at 33, 34 and 35 weeks corrected age.
- compare breastfeeding rates and duration between groups using the conventional teat (Control group) or Calmita teat (Intervention group) for supplemental feeds during the transition to full breastfeeding

What is the nature of the study?

Mothers and babies in our study will participate in a randomised controlled clinical research project within the Neonatal Unit at King Edward Memorial Hospital (KEMH). We will monitor babies' coordination of sucking, swallowing and breathing, their heart rate and oxygen saturation levels, and the effects of breast fullness, nipple shield use and different bottle teats on breastfeeding outcomes. We do not know if one type of bottle teat is better than another with regard to establishing full breastfeeding. To find out we need to compare two different bottle teats. We put study participants into one of two groups and use a different bottle teat for each group. The results are compared to see if one is better. To try to make sure that the groups are the same, each participating mother-infant pair is put into a group by chance (random).

At each study breastfeed, mothers will complete a short questionnaire that rates the baby's breastfeeding efforts.
Mothers will be asked to record their breast expression and breastfeed volumes over one 24hr period at 33, 34 and 35 weeks CGA. Mothers also have the option of providing very small samples of breastmilk (<1.0mL) collected before and after breast expressions and breastfeeds so that breast fullness can be more accurately estimated.

What will the study tell us?

This research project will provide new information about the growing preterm baby's developing sucking strength, coordination of sucking, swallowing and breathing, and the effects of nipple shields and bottle teats on breastfeeding success. This information will be used to determine the normal development of breastfeeding behaviours in preterm babies and will guide the appropriate use of nipple shields and bottle teats to support the establishment of breastfeeding.

It is anticipated that the results of this research project will be published and/or presented in a variety of forums. In any publication or presentation, information will be provided in such a way that individual mothers and babies cannot be identified. A summary of study results can be made available to participants on request; it is expected this will not be available until the conclusion of the study, anticipated to be end 2016.

Who is carrying out the study?

Principal Investigator: Professor Karen Simmer, Professor of Neonatology, Director, Neonatology Clinical Care Unit, KEMH

Local contact people

Sharon Perrella, Research Associate	0410 056 779
Associate Professor Donna Geddes	6488 7006

Hartmann Human Lactation Research Group, University of Western Australia (UWA)

This study is sponsored by the Telethon and Women and Infants Research Foundation.

What you will be asked to do if you decide to take part?

We would like to weigh your baby immediately before and after every breastfeed ('test weigh') during the study period so that the volume of milk taken at the breast can be measured. When a full feed has not been taken, the test weigh measurements will be used to calculate the volume of top up feed to be given.

For this study, babies will be allocated to use a conventional teat (Control Group) or vacuum controlled Calmita teat (Intervention Group) for use when the breastfeeding mother is not available for scheduled suck feeds, and for top up feeds that are given via a bottle. Intervention group babies will use the Calmita starter teat and change to the Calmita advanced teat when they have consistently sucked full feeds using the Calmita starter for 48-72 hours. Intervention group families will be provided with Calma teats at transfer or discharge from KEMH SCN so they can continue using a vacuum controlled teat for top up bottle feeds until the baby is full breastfed.

We would like to monitor up to 5 breastfeeds during your baby's stay in the nursery. Monitoring of a breastfeed includes

- continuous measurement of your baby's heart rate and blood oxygen level using your baby's usual pulse oximetry equipment that is placed on the hand or foot using a soft Velcro strap

- continuous measurement of your baby's breathing through placement of one soft elasticised band around your baby's chest and one around the abdomen
- measurement of the strength of your baby's suck through taping of a very fine soft pliable plastic tube to the breast, and
- ultrasound observation of your baby's tongue movements during breastfeeding using a small ultrasound probe under your baby's chin during breastfeeding.
- Mother's completion of a short questionnaire to assess the baby's breastfeeding effort

We ask that when the infant is 33, 34 and 35 weeks corrected age, mothers record their 24hr milk production volume by recording all volumes of expressed milk and volumes that the baby takes from the breast (measured through test weighing). Mothers also have the option of providing breastmilk samples <1.0mL (collected before and after each feed and expression during the 24hr period) to determine fat content. This provides a more accurate estimate of the volume of milk available in the breast. Re-identifiable samples will be transported to UWA for frozen storage, and analysed at a later date. Any remaining milk will be kept in frozen storage until publication of results in a peer reviewed scientific journal.

The study requires for mothers to be available for a daytime breastfeed on a weekday; once at 33 weeks and up to 2 times at 34 weeks and 35 weeks corrected age or at discharge from KEMH.

At 33 weeks and 35 weeks one breastfeed will be monitored under the usual conditions for each mother and baby.

At 34 weeks we would like to monitor breastfeeds under two different conditions:

- Breastfeeding on a full breast (no expressing in the previous 3 hours)

- Breastfeeding on an 'empty' breast (breast was expressed within the previous hour)

At 35 weeks if the mother is already routinely using a nipple shield we will ask her if we could also monitor a breastfeed without a nipple shield.

At each week of the study, we would also like to monitor one bottle feed with your baby's allocated (conventional or Calmita) teat. For babies remaining in KEMH Neonatal Unit after 35 weeks corrected age, mothers will be invited to continue weekly monitored breastfeeds until discharge or transfer.

Following discharge home, mothers will be invited to return to KEMH for one monitored breastfeed when the baby reaches term corrected age – if the baby was born <32 weeks gestation and it is convenient for the family, this may coincide with the baby's term follow up appointment at KEMH.

Mothers will be contacted by telephone when the baby is 2, 6, 12 weeks corrected age to enquire about breastfeeding. Follow up will end when the baby reaches 3 months post term, or when the infant has weaned from breastfeeding, whichever occurs first.

Development of Breastfeeding Skills in Preterm Infants

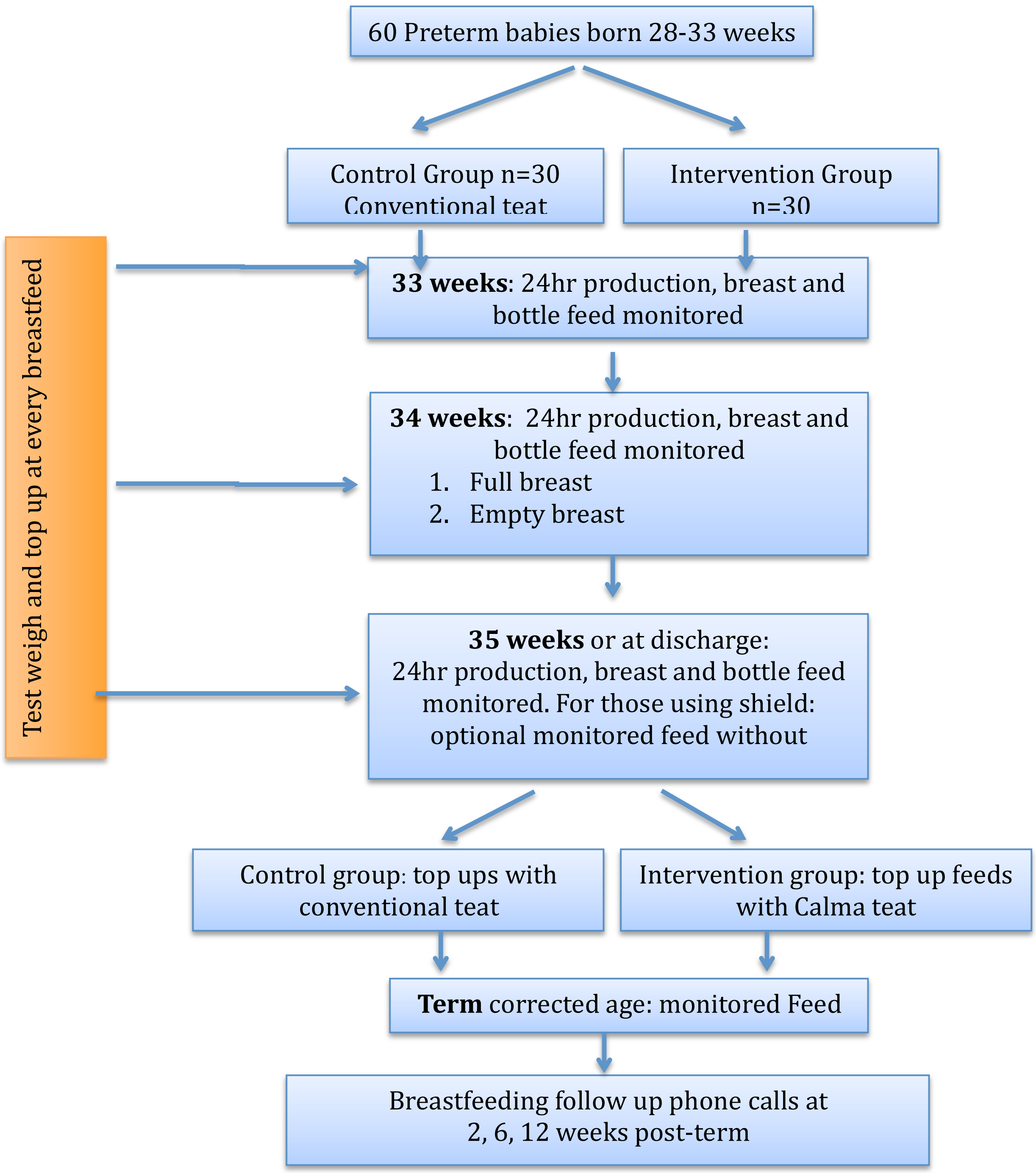

What are the benefits and risks of taking part?

We cannot guarantee or promise that your or your baby will receive any benefits from this research. However, potential benefits to the mother may include feedback about the baby's developing breastfeeding skills such as sucking strength and volumes transferred at the breast. Monitoring of milk production will provide mothers with information about the adequacy of their supply, and the mother's efforts to provide breastmilk and to breastfeed the baby will be encouraged and supported. Researchers may assist with identification of any breastfeeding or lactation issues and arrange referral to appropriate resources.

We do not expect that there will be any risks to babies participating in the study. Some of the study monitoring is already routinely performed in the nursery such as heart rate and oxygen level monitoring. The method used to measure babies' breathing rates (Respiratory Inductive Plethysmography) involves having two soft bands placed around the baby's stomach and chest. To measure the baby's tongue movements during feeding a small ultrasound probe is gently held under the baby's chin. Ultrasound is already routinely used in the nursery. The strength of the baby's suck is measured using a very fine plastic tube that sits alongside the mother's nipple or bottle-feeding teat. This does not interfere with feeding. No negative effects have been observed with any of these monitoring methods.

There are no foreseeable risks to the infant as oxygen saturation and heart rate monitoring are routinely performed in the neonatal nursery without complications. Monitoring of respiratory rate using Respiratory Inductive Plethysmography, and of tongue movements during feeding using ultrasound are both non-invasive methods with no known adverse effects. Intraoral vacuum measurements require a very fine plastic tube to be taped to the mother's breast – this does not extend beyond the nipple during breastfeeding. Young babies do not appear to be aware of the presence of the tube, and no adverse effects have been noted.

The information gained from this study will assist in guiding the establishment of breastfeeding in preterm babies, and in aiding the transition to full breastfeeding through appropriate use of lactation aides such as nipple shields and bottle teats.

Do I have to take part?

Your participation is voluntary. If you do not wish to take part, you do not have to; your treatment at this Hospital will not be affected in any way. If you do decide to take part, you will be given a copy of this Participant Information Sheet to keep. If you decide to take part but later change your mind, you are free to withdraw from the project at any stage.

What are the costs?

There are no additional costs associated with participating in this research project, nor will you be paid. All monitoring and tests and vacuum-controlled teats (for those allocated to that group) required as part of the research project will be provided to you free of charge.

What about my privacy?

By signing the consent form you consent to the research staff collecting and using information provided by you, and from your baby's health records for the research project. Any information obtained in connection with the research project that can identify you or your baby will be kept confidential, with paper records kept in a locked cabinet at the University of Western Australia. Electronic data and milk samples will be re-identifiable (coded) and electronic data will be stored on a laptop computer in password-protected files. Only the researchers will have access to the data. Data will

be stored for seven years after completion of the study and will be destroyed at the end of the storage period.

What will happen to information about me?

Information about your baby may be obtained from health records held at KEMH for the purpose of this research. By signing the consent form you agree to the study team accessing health records if they are relevant to your participation in this research project. Any information obtained in connection with this research project that can identify you or your baby will remain confidential. Your information will only be used for the purpose of this research project and it will only be disclosed with your permission, except as required by law. It is anticipated that the results of this research project will be published and/or presented in a variety of forums. In any publication and/or presentation, information will be provided in such a way that you or your cannot be identified.

Who has approved the study?

Women and Newborn Health Service Human Research Ethics Committee.

Who to contact for more information about this study:

If you would like any more information about this study, please do not hesitate to contact one of the research team. They are very happy to answer your questions.

Name	Title	Contact number
Sharon Perrella	Research Associate	0410 056 779

Who to contact if you have any concerns/complaints about the study or its organisation?

*If you have any concerns or complaints regarding this study, you can contact the **Director of Medical Services at KEMH** (Telephone No: (08) 9340 2222). Your concerns will be drawn to the attention of the Ethics Committee who is monitoring the study.*

What to do next if you would like to take part in this research?

If you would like to take part in this research study, please read and sign the consent form provided.

THANK YOU FOR YOUR TIME AND CONSIDERATION

Appendix C: Study Information

FORM OF CONSENT

PLEASE NOTE THAT PARTICIPATION IN RESEARCH STUDIES IS VOLUNTARY AND SUBJECTS CAN WITHDRAW AT ANY TIME WITH NO IMPACT ON CURRENT OR FUTURE CARE.

I .... have read
Given Names Surname

the information explaining the study entitled

Development of Breastfeeding Skills in Preterm Infants

I have read and understood the information given to me and consent to

……………………………………………………………………………………...
(insert baby's name)

participating in the study. Any questions I have asked have been answered to my satisfaction.

I understand I can withdraw my baby from the study at any stage and withdrawal will not interfere with routine care.

I agree that research data gathered from the results of this study may be published, provided that names are not used.

Dated .... day of .... 20 ....

Signature ....

I, ...have explained the above to the
(Investigator's full name)

signatory who stated that he/she understood the same.

Signature..

Appendix D: Case Report Form, Master

addressograph sticker

(complete at recruitment)

Mother's name:

Mobile no:

e-mail: (if unable to contact via phone)

Mother's birth date:

☐ Mother has requested study report

Baby's name: Male / Female

Date of birth: Birth gestation: Birth weight: SGA/AGA/LGA

Ventilation days: CPAP days: IVH grade: Sepsis:

SCN Study schedule (B=breast, NS= nipple shield, record dates, teat type)

CGA	bottle	B + NS	B - NS	Full B	Empty B
birth gest =					
33/40					
34/40					
35/40					
36/40					
37/40					
38/40					
CGA	date				
		first sucked a full feed			
		first suck all feeds over a 24hr period			

Date and CGA at transfer / discharge:

Calma teats given? ☐ Yes ☐ NA

Breastfeeding background data (complete at recruitment)

"Please tell us about your breastfeeding experience and plans"

Have you breastfed any previous babies?

1. no
2. yes – I breastfed for ___________ days / months (range if > 1 baby breastfed)

For how long do you plan to breastfeed this baby? _____________ months

How important is BF to you?

1. Not at all important
2. Not important
3. Important
4. Very important

Thinking back over the last 24 hours, what is the total amount of milk you have expressed? _______________ mL (baby is ______ days old)

addressograph sticker

Appendix E: Case Report Form Monitored feed

Date:

CGA: 33 34 35 36 Other:

Postnatal days: Naked weight: _______________g

Prescribed feed volume: _______mL ☐3hrly ☐2hrly _____IGT : ___suck

Breastfeed

Breast fullness ☐Full (last expressed ≥ 3hr) ☐Empty (last expressed ≤1hr)

Nipple shield ☐ No ☐ Yes size:

Bottle feed

☐ Calmita starter ☐ Calmita advanced ☐ Conventional

Pre feed weight: Pre feed milk sample ☐

Post feed weight: Post feed milk sample ☐

Total volume transferred:

Heart rate range:

SaO_2 range:

☐ Resp plethysmography

☐ Intraoral vacuum

☐ PIBBS completed by mother PIBBS score: ___________

☐ 24hr production and milk samples collected Result _____________mL

Total number of feeds in last 7 days: __________

Total number of oral feeds: __________

Of these, total breastfeeds: __________

Adverse events – details eg. bradycardia, apnoea, interventions required

Notes:

Appendix F: Case Report Form Follow up interview

addressograph sticker

Corrected age (weeks): 2 6 12

Please tell me is (baby) still breastfeeding or receiving your breastmilk?

NO → Can you tell me when (baby) last received a breastmilk feed?
(record date and corrected age)

What were your reasons for weaning? ____________________________________
Thank for participation in study → no further follow up required

YES → I would like to ask some questions about how your baby is feeding.

Thinking back over the last 24 hours, how many times has your baby fed? __________
How many of these were breastfeeds? ___________
Do you use a nipple shield when breastfeeding? Yes No
(if not fully breastfeeding…)
How many of the feeds were bottle-feeds? ___________
Of those, how many were breastmilk feeds?
How many full feeds were given by bottle?
How many top up feeds were given by bottle?
What type of teat/s do you use to bottle feed?
Are you having any difficulties with feeding?

If any questions or concerns are raised with regard to feeding, refer mother to
SCN home visiting nurse (if home visiting is still being provided)
KEMH Breastfeeding Centre
GP as appropriate
ABA as appropriate

Thank you for taking the time to answer these questions today. I will call again when your baby is ____ weeks corrected age.
